# Understanding patient experiences in a motivational interviewing intervention to improve whole-person lifestyle among individuals with hypertension or type 2 diabetes: a qualitative focus group study

**DOI:** 10.1080/17482631.2021.1978373

**Published:** 2021-09-22

**Authors:** Amanda T. Sawyer, Kim McManus

**Affiliations:** AdventHealth, Research Institute, Orlando, FL, USA

**Keywords:** Diabetes, hypertension, qualitative research, wellness

## Abstract

**Purpose:**

This qualitative focus group study aimed to determine how participants responded to a motivational interviewing intervention and to further explore how it impacted whole-person lifestyle of participants with hypertension or type 2 diabetes.

**Methods:**

Twenty participants attended one of five focus groups. A trained researcher led the one-hour focus groups using a semi-structured question guide. Responses were coded using thematic analysis and were then aggregated into six themes.

**Results:**

The following six themes emerged most consistently: (1) the importance of a coach who can connect meaningfully with participants; (2) appreciation of the whole-person approach; (3) the power of “choice” in making health behaviour changes; (4) the effectiveness of goal setting and accountability; (5) the desire for increased contact and follow-up; (6) overall positive experience with mixed clinical results.

**Conclusion:**

Focus group themes highlighted that this intervention may empower individuals to feel confident in their choices and attain their goals during their health and wellness journey.

## Introduction

In the USA, up to half of premature deaths are caused by behavioural and other preventable factors (Committee on Population; Division of Behavioral and Social Sciences and Education; Board on Health Care Services; National Research Council; Institute of Medicine, 2015). Cardiovascular disease (CVD) is the leading cause of premature death in the USA (Chen et al., 2019). The prevalence and associated medical costs of CVD are expected to increase by 2030 (Heidenreich et al., 2011). Policy efforts to prevent CVD include mass media campaigns, food pricing strategies related to taxation and subsidies, school procurement policies, and worksite wellness programmes (Afshin et al., 2015). Effective behavioural prevention strategies are also needed to reduce the growing burden of CVD. Considering the links between individual health behaviours and cardiovascular health, interventions that focus on health behaviours, such as diet, physical activity, and smoking, may improve associated health outcomes and reduce costs to the health care system (LeFevre, 2014; Morton et al., 2014; Sawyer et al., 2020; Spring et al., 2013).

The “Five A’s” is a framework for health behaviour change counselling during a medical visit, which includes five counselling steps that a provider can complete in several minutes: (1) assess the risk behaviour, (2) advise change, (3) agree on goals and an action plan via shared decision making, (4) assist with treatment, and (5) arrange follow-up (Ammerman et al., (2002).; Spring et al., 2013). Motivational interviewing is a specific type of patient-centred counselling that leverages this framework and has been used to foster health behaviour changes through self-reflection (Hettema et al., 2005; Miller & Rollnick, 1991; Spring et al., 2013). It has been conducted with individuals to prevent unhealthy behaviours and promote healthy behaviours (Frost et al., 2018). Systematic reviews have shown the positive effects of motivational interviewing on weight loss among adults who are overweight or obese (Armstrong et al., 2011; Barnes & Ivezaj, 2015), as well as the improvements in other metabolic risks, such as triglyceride levels, waist circumference, and systolic blood pressure (Lin et al., 2014).

A whole-person health intervention was developed based on CREATION whole-person health elements to address behaviour change in individuals diagnosed with hypertension or type 2 diabetes recruited from a primary care office (Sawyer et al., 2020). CREATION is an acronym that stands for the eight whole-person health elements of choice, rest, environment, activity, trust, interpersonal relationships, outlook, and nutrition. The definitions of these elements (Table I) were established through a literature search and focus groups of administrators, clinicians, patients, and families (Sawyer et al., 2020). These definitions were used as the foundation of this intervention.

**Table I.** Definitions of creation whole-person health elements Sawyer et al. (2020)

Element	Definition
Choice	Choice is a complex element that is impacted by emotional, spiritual, physical, and environmental factors. The other elements influence choices; in turn, those choices play a part in our habits and behaviours.
Rest	Rest includes physical rest, cognitive breaks, and emotional and spiritual restoration.
Environment	Environment includes all components of our physical surroundings and resources, which includes living space, safety, workplace, food, healthcare, transportation, and recreational space.
Activity	Activity integrates physical, mental, and spiritual activities into our daily lives.
Trust	Trust encompasses connections with God, faith, loved ones, colleagues, and those who have been entrusted to lead.
Interpersonal Relationships	Interpersonal relationships involve the support, connection, and belonging shared with family, friends, colleagues, and faith communities.
Outlook	Outlook involves the sum of attitudes, perceptions, and psychological health and influences daily life and relationships.
Nutrition	Nutrition is nourishment for the body and the source of energy for the mind. Healthy food is essential to wellness and disease prevention.

This tailored intervention focused not only on physical wellness components, such as diet and exercise, but also on wellness components related to the mind and spirit. Using motivational interviewing techniques, a programme specialist, who was a registered nurse, prompted participants to explore how to make lifestyle changes, such as healthier eating habits, increased physical activity, and more mindful decision-making. In this role, the programme specialist, also called a coach, served in an auxiliary role to the physician.

The quantitative study findings showed statistically significant changes in BMI and waist circumference between the intervention and control groups at the 6-month follow-up (Sawyer et al., 2020). Also, the proportion of high total wellness scores, measured through self-reported data with the Wellness Evaluation of Lifestyle (WEL) (Myers et al., 2004), in the intervention group increased after the whole-person lifestyle intervention (Sawyer et al., 2020). A better understanding is needed about how focusing on whole-person health elements through motivational interviewing in the primary care setting may influence behaviour change and wellness. This descriptive qualitative focus group study with an inductive approach aimed to determine how participants responded to the motivational interviewing intervention and to further explore how it impacted whole-person lifestyle of participants with hypertension or type 2 diabetes.

## Methods

### Participant selection

Inclusion criteria for participants in the whole-person health intervention were diagnosis of type 2 diabetes, fasting glucose >125 mg/dL, or HbA1c >6.5% and/or diagnosis of hypertension and age 18 years or over. Exclusion criteria has previously been described (Sawyer et al., 2020). Informed consent was obtained from all participants prior to any study procedures.

After completing this intervention, all participants were invited during their 6-month visit to attend a focus group. Interested participants self-selected into a convenience sample. A total of 20 participants attended one of five focus groups scheduled over nine months. There was an equal number of male and female focus group participants. Each focus group consisted of participants of both genders and both diagnoses of hypertension and type 2 diabetes. Participants who attended a focus group received a gift card valued at $20 for their time.

### Setting

The research team held the focus groups in a private conference room in the hospital affiliated with the primary care office. Only the participants and researchers were present.

### Data collection

A Ph.D. research scientist, trained and experienced in focus group facilitation, led the one-hour focus groups. She had no relationship with the participants prior to the focus groups. At the beginning of each meeting, she introduced herself and explained the reasons for the focus groups. Then, a semi-structured question guide was utilized to ask participants the following prompts: why they agreed to participate in the study; what changed for them because of their participation, and why it changed; what they would tell friends or family about the programme; and what they would change about the programme. Once the data reached saturation, the groups concluded. The facilitator recorded the focus groups on audiotapes. A second member of the research team also took handwritten notes during each focus group. Transcripts were not returned to participants for comment or correction. Focus groups were conducted rather than individual interviews because this method allows qualitative data to be collected in a time-efficient manner, while also providing an opportunity for participants to discuss the shared experience and engage in helpful support and networking.

### Data analysis

Using Braun’s model, thematic analysis was used to code responses by keywords, which were then aggregated into six themes (Braun & Clarke, 2006). The focus group facilitator assigned participant responses to categorical codes, line-by-line, to analyse patterns of relationships. A constant comparison technique was used to identify similarities and differences throughout the data. The following coding techniques were used to analyse transcriptions: (1) open coding to segment the data into preliminary categories based on similarity; (2) axial coding to group the categories into themes that provide new ways of seeing and understanding the phenomenon under study; and (3) selective coding to integrate the categories and themes to articulate a coherent theory of the phenomenon under study. After coding was completed, the study’s principal investigator reviewed all transcriptions and assessed the coding to confirm alignment. The principal investigator’s assessment resulted in no modifications to the original coding.

### Ethical approval

This study was reviewed and approved by the AdventHealth Institutional Review Board (IRBNet # 878,401). This study was guided by the ethical principles of autonomy, beneficence, non-maleficence, and justice, and it fulfilled the research requirements of consent, confidentiality, and safety. The study team maintained confidentiality for participation and data collection, provided both verbal and written information about the study to participants, and ensured that they understood that they could withdraw from the study at any time without explanation.

## Results

Across the five focus groups, participants shared their unique perceptions of their experiences. The intervention’s whole-person approach to wellness encouraged individual customization and allowed participants to engage in the intervention in the context of their own needs and situation. Various aspects of the programme appealed differently across the focus group participants, but the following six themes emerged most consistently (Table II): (1) the importance of a coach who can connect meaningfully with participants; (2) appreciation of the whole-person approach; (3) the power of “choice” in making health behaviour changes; (4) the effectiveness of goal setting and accountability; (5) the desire for increased contact and follow-up; (6) overall positive experience with mixed clinical results. Each theme is documented below with a brief overview, followed by the participants’ own words to illustrate their perceptions.

**Table II.** Qualitative themes and categories

Theme	Categories
1) Importance of a coach who can connect meaningfully with participants	Role critical to success Flexible to meet individual needs Judgement-free
2) Appreciation for the whole-person approach	Integration of multiple approaches Relationship between physical and mental wellness Customized resources
3) Power of “choice” in making health behaviour changes	Choice to participate in study Choice to avoid medications
4) Effectiveness of goal setting and accountability	Manageable goals Meaningful goals Accountability as a driver
5) Desire for increased contact and follow-up	Benefits of structured follow-up Continuity with support network
6) Overall positive experience with mixed clinical results	Pride in improvements Recognition of ongoing journey to wellness

### Theme 1: importance of a coach who can connect meaningfully with participants

Focus group participants frequently spoke about the influence of their coach on their motivation and goal achievement. Many agreed that working with a coach who provides direction and encouragement, without judgement and with the flexibility to meet each individual’s needs, was one of the strongest influences on their perceived success. Although one programme specialist led this intervention, additional programme specialists may ensure better compatibility between the coach and participant. Focus group responses strongly confirmed the coach’s role in participants’ success.

*[The programme specialist] was always there to encourage you, and it was refreshing because it was something to look forward to. Talking about life, how to interact, being active … my daughter plays lacrosse, and [the programme specialist] suggested that I practice with her. Now I do that about twice a week. It surprised me how much the programme helped not only myself but those around me.*

#### Direction and encouragement

Participants emphasized that they received help in finding the right combination of behaviours to develop healthier lifestyles. They appreciated creative ways to incorporate healthy habits into their lives in a way that suited their individual needs and situations.

*[The programme specialist], the moderator, was very personable and easy to talk to. She was a guide pointing me in directions I have not thought of – mainly exercise and diet. She let me focus on it. A lot of what I heard, I have heard before. It was the direction that I got out of the study.*

#### Non-Judgemental

Some participants spoke about feelings of shame they had experienced in the past for failing to meet their health goals. They partly attributed the coach’s effectiveness to the ability to help participants recover from setbacks.

*I never felt guilty about anything. She was never judging … She made it comfortable – no commitment – we could always drop out.*

*That is something I liked about the programme – it is not judgmental. She always changed something to make it positive. After many years of struggling with weight loss, you beat yourself up. Being able to let go of that …*

*When I mess up, I hear [the programme specialist’s] voice – God gives you grace, give yourself some grace.*

References to shame resurfaced in conversations about the relationship between physical and mental wellness. Focus group participants attributed their improved awareness of the interaction among mind, body, and health to their participation in the programme. They also compared the intervention to therapy. However, some participants noted the perceived stigma of mental health treatment.

*Health coach removes the stigma (compared to meeting a therapist).*

*I am too proud to go to a therapist, but a health coach is okay.*

Participants were now aware of the connection between mind, body, and spirit through the intervention’s whole-person approach and the access to a “toolbox” of solutions.

### Theme 2: appreciation for the whole-person approach

Several participants spoke about their past experiences with intentional approaches to improving their health, often with little lasting success. These approaches typically stressed diet, exercise, or both. Integrating these crucial components with strategies for mental and spiritual wellness resulted in a different experience for participants.

*When you go through it from different perspectives, suddenly it will be so much easier because you understand how all of these different areas interact and can really support you. What you need might be some support in the spiritual side, or maybe it is about your choices. It is like a jigsaw puzzle.*

*I liked the combination of all the aspects and the addition of spirituality into the mix. It involves all aspects of life. Even if you’re not completely successful in all the areas, you still have the success in certain areas. I always felt as I was leaving the session that I have learned so much and that I am loved.*

Most participants related something from their personal situations during the focus group and reported ways their coach attempted to customize their goals and engagement with programme resources.

#### Customized resources

Some participants expressed the effectiveness of the educational materials they received in the programme, while others admitted to never looking at them. While some participants were committed to journaling, others never tried to use that tool. For the most part, participants expressed the effectiveness of access to multiple resources and the freedom to engage in those that best suited them.

*As an older person who has been through programmes, who has joined a programme and lost 50 lbs. twice, I found the book really wonderful to read. Maybe it is not new information about food and exercise, but I loved the perspective, the holistic perspective. I felt that I did have something new because it is looking at yourself as a whole person … a new framework.*

*You never know how many aspects are related to your weight (happy at home, eating better because it is the right thing to do, eating healthier). It is a lifelong thing … We talked about relationships at work/at home. We would talk about things that were encouraging … I would love to go and meet with [the programme specialist], not just my weight and blood pressure because the things we talk about would influence these things.*

A whole-person approach to health and wellness considers the interrelatedness of our choices, including the overarching choice to take responsibility for self-care. Focus group participants reported increased awareness of how their choices influenced their health.

### Theme 3: the power of choice

One of the eight CREATION elements is choice, which is an intentional decision to take a possible course of action. The foundation of a healthy, balanced lifestyle is the ability to make healthy choices and exert control over one’s wellness. Many focus group participants expressed their understanding of and appreciation for the power of choice.

*I think the biggest thing for me that I took away from the programme was ‘choice.’*

*Sometimes you already know those things, but suddenly they make sense that comes so naturally. You find yourself making good choices without really thinking about it.*

*You will learn a lot about yourself and the choices that you make and why you make these choices.*

*For me, it was about choice. When you make a choice, that is when the change starts.*

*My emotional response was how [the programme specialist] managed the programme. The kind of logical side looking at all the parts of it, it boils down to ‘choice.’*

Some focus group participants talked about choice in the context of agreeing to participate in the study. For many, the choice was driven by mounting concerns about their ongoing health issues.

#### Avoidance of medications as a motivator

Participants were referred to the programme by their physician, often during treatment for high blood pressure, diabetes, or other health complications. For many of these participants, a strong motivation was to avoid dependence on medications.

*I was 40 pounds overweight, with diabetes and high blood pressure, taking meds that I wanted to get off … I thought this programme would be an extra push.*

*The goal is to bring my numbers down somehow. I do not want to take so many pills now.*

### Theme 4: the effectiveness of goal setting and accountability

There was agreement about the effectiveness of being held accountable on a regular schedule as motivation to work towards health goals. Participants unanimously reported that goals were motivators only when they were manageable and meaningful.

#### Manageable goals

Some participants discussed feeling overwhelmed by the gap between where they were and where they wanted to be with their health. Others described past experiences of unrealistic goals leading to failure and ultimate abandonment of efforts to improve. The programme was useful for these participants because they agreed with their coach on small goals with manageable timelines.

*When we set the mini-goals, that made me motivated. I was 190, and I lost 20 pounds. It works better when you set goals that you are comfortable with.*

*When you have a lot of weight to lose, it really is quite overwhelming. Setting the mini-goals is helpful.*

*[The programme specialist] was very encouraging – you know, baby steps. So many things now are quick fix, but with this programme, they want to extend this out to a life-changing thing. Not like going to the gym for a beach body, and then not going in the winter.*

#### Meaningful goals

For many participants, part of the attraction of the whole-person approach is flexible intervention options that can be tailored to each individual. For some, that means creating goals that reflect personal circumstances and opportunities. Goals perceived as meaningful were more likely to be “owned” by the participants, increasing their control over and responsibility for maintaining a balanced and healthy lifestyle.

*My goal for the future is that I want to be running around with my grandkids. For that, my goal was to lose weight. I would prefer that my insurance would pay for these kinds of programmes than getting my blood pressure readings.*

*If I step back and look at it, the brilliance of the programme is that you’re accountable to yourself, not [the programme specialist]. These are my goals; she is just transcribing them.*

Whatever goals individual participants were working towards, most reported the importance of being held accountable for those targets by their coach.

#### Motivation through accountability

Some participants reported accountability to their coach as a motivator. Others said they felt accountable to a family member or someone else to meet the goals they were setting in the programme. Some participants suggested establishing peer accountability through an “accountability partner,” in addition to the coach, to extend the intervention’s positive effects.

*For me, it was the accountability. Knowing I had to talk about my progress kept me on track.*

*Importance of checking in on a regular basis … knowing I would be seeing [the programme specialist] in two weeks.*

*Having an accountability partner outside of the programme or someone to come in with you. Getting a group together and talking about what I am experiencing, getting encouragement from each other.*

*It was important to see someone on a regular basis, and it was less threatening than therapy.*

*I would like to go more often. There would be more accountability if I was seeing [the programme specialist] more often.*

Accountability was reported as a driver for regular contact with a coach or other supportive partner. Participants also expressed a desire for extended support and communication for other reasons.

### Theme 5: the desire for increased contact and follow-up

While some participants reported appreciation for the individualized attention and goal setting, others perceived their experience differently. Some participants experienced the intervention as too regimented, feeling rushed through topics that interested them most. Some reported the time they spent on the computer to complete assessments as a distraction and barrier from more beneficial coaching conversations.

*We went into the reasons why we eat, but then we would have to go to a different topic because of the schedule … There is such a strict timeline that you could not go any further.*

*The only thing that could probably be improved is possibly the amount of time spent on topics. It felt like it was rushed.*

*Conversation time was not a lot. The [surveys] took up a lot of time. Maybe you had 15 minutes or even less, which is not enough time to develop a personal relationship. You need more than that.*

The desire for increased contact included more time for customized coaching and opportunities for follow-up support upon completion of the intervention.

#### Potential benefits of structured follow-up

Many participants expressed the potential benefits of follow-up support, such as extended time with the coach and opportunities to develop accountability partners. Some noted the benefit of sharing their thoughts and feelings with other participants during the focus group.

*It would be nice to have the option to continue meeting once every six months and then start the programme again if 1-on-1 sessions are needed.*

*I wish there was a way to continue once a week with an accountability partner.*

*What I wish [the hospital] had – A place where someone like me could go with a gym with people who understand weight loss and understand physical limitations.*

*The people in the programme should meet more often than only the focus group for more support.*

Participants’ reports of positive experiences with the intervention and their interest in continuing whole-person lifestyle interventions were consistent with their affirmations of overall positive experiences, sometimes despite falling short of their goals.

### Theme 6: overall positive experiences and mixed clinical results

Some participants were proud to report the improvements to their health they attributed, at least in part, to their participation in the study. These improvements include lower HbA1c, blood sugar levels, weight, cholesterol, and blood pressure.

*I started hobbies using my hands to keep them busy. HbA1c went down from 6.2 to 5.8 because I am watching all this stuff.*

*My endocrinologist is so happy because my blood sugar levels are definitely lower and went down. They are very stable now, and I have lost weight.*

*I lost 40 pounds on the programme. I’m able to spend time on hobbies. I no longer have plantar fasciitis because of the weight loss. My outlook was helped by refocusing on my hobbies.*

*My cholesterol went from 190 to 158 on this programme.*

*My blood pressure has come down. I am 32, and I have had it all my life. I played college football, and even when I was in the best shape of my life, I had high blood pressure. I went from 160/98 down to 135/82 during the programme.*

*My blood pressure was consistently in the 150s and high 90s, now in 120s. I attribute a lot of that to being in the programme with [the programme specialist].*

Not every participant reported they had met the goals they established in the programme, or that they had been able to sustain positive gains. However, nearly every participant reported feeling they were still on the journey they started with their wellness coach.

*I knew a lot of things, but it is no longer just a diet. This programme helped me make a change in my approach to eating … a permanent change in the way I approach food … now I walk away from a meal before finishing … I have changed habits that I have had since I was 4 years old … we were not allowed to leave food on our plates.*

## Discussion

Given the complex nature of behaviour change, a whole-person approach can encourage individuals to address the mental, spiritual, and physical aspects of their life. This qualitative study explored how participants responded well to the components of this motivational interviewing intervention and found goal setting and accountability from a coach to have a positive impact on participants’ whole-person lifestyle behaviours.

First introduced in adult health addiction services in Miller (1983), motivational interviewing has become an intervention used for physical health, especially to encourage behaviour change among individuals diagnosed with chronic conditions. For example, motivational interviewing has been utilized in efforts to improve outcomes related to the management of type 1 diabetes, type 2 diabetes, and obesity in both adult and paediatric populations (Christie & Channon, 2014). It has also been used to increase physical activity self-management for adults with type 2 diabetes (Soderlund, 2018).

This whole-person lifestyle intervention supported health behaviour modification through motivational interviewing to improve not only physical health indicators, but also mental and spiritual wellness indicators. Focus group responses provided a collective view of the intervention that informs the interpretation of the quantitative study finding of an increased proportion of high total wellness scores on the WEL (Sawyer et al., 2020). Participants discussed aspects of the intervention that reflected the dimensions of mental and spiritual wellness on the WEL (Myers et al., 2004). Many of the WEL scales align with the CREATION elements, and the two scales of sense of control and realistic beliefs are related to motivational interviewing. A sense of control involves beliefs about one’s competence, confidence, and mastery (i.e., “I can”), as well as the belief that one can usually achieve their goals (Myers et al., 2004). With a sense of control, a person can make choices through imagination, knowledge, and skill. It also involves planning one’s life and being able to express one’s needs directly. Realistic beliefs allow a person to process information and perceive reality accurately. An individual with realistic beliefs can identify logical and rational thinking and control the “shoulds,” “oughts,” “dos,” and “don’ts” (Myers et al. 2004).

Focus group data confirmed that improvements in wellness scores may be partially attributed to the counselling participants received as part of motivational interviewing. The importance of flexible and non-judgemental interactions with their coach and the desire for continuing structured support emerged as key themes. The coach’s role was also integral to the other themes: providing direction in goal setting, finding whole-person approaches to individual circumstances, and instilling pride and commitment to participants’ personal wellness journeys. Social support has been associated with better self-care in patients with diabetes (Song et al., 2017; Van Dam et al., 2005), as well as favourable health outcomes (Van Dam et al., 2005). Additionally, goal setting and accountability in diabetes care and self-management are supported in the literature as recommended components (Fredrix et al., 2018; Miller & Bauman, 2014). To encourage long-term goal setting and accountability, it may be beneficial to enable participants to develop accountability partners.

It is important to note that choices regarding health and wellness are not made independently of an individual’s circumstances. The elements of Trust, Interpersonal Relationships, Outlook, and Environment influence one’s health-related choices and vice versa. Similarly, one’s health-related choices impacts the behavioural elements of Rest, Activity, and Nutrition and vice versa. Through a whole-person approach, participants gain more understanding of choice, which is taught and encouraged through motivational interviewing. They also learn about the link between their mental, spiritual, and physical wellness. For these reasons, addressing whole-person health elements through motivational interviewing in the primary care setting may encourage patients with hypertension or type 2 diabetes to set attainable goals and improve choices related to their health behaviours.

This study has some limitations. Although these focus group participants cannot be considered representative of all study participants, the knowledge generated through this qualitative study may be useful in other clinical settings and populations when considering motivational interviewing (Tracy, 2010). Thematic analysis is an interpretive methodology that is subject to investigator bias. The investigator attempted to minimize bias through critical reflection intended to surface and challenge assumptions and experiences that may result in bias. Participants’ ability to choose whether they wanted to participate in a focus group introduced self-selection bias into this qualitative focus group study. Although these participants had mixed clinical results, all were compliant with intervention session attendance, which may reflect their positive experiences in the intervention. Although focus group participants appeared to be responding openly and without discomfort, they responded in the presence of a small group of others, without anonymity and subject to perceived peer pressure or discomfort with responding openly.

### Conclusion

This qualitative focus group study of a whole-person lifestyle intervention affirms the value of providing a whole-person intervention with motivational interviewing to empower individuals to feel confident in their choices and attain their goals during their health and wellness journey. Future research is needed to explore a feasible method of allowing primary care physicians to make referrals to a motivational interviewing coach who can be embedded in a large medical group or health park setting for ongoing support. This type of motivational interviewing intervention can be used to help patients with type 2 diabetes and/or hypertension improve their mental, emotional, social, and spiritual wellness alongside their physical health.
